# Editorial: Characterizing and improving traits for resilient crop development

**DOI:** 10.3389/fpls.2023.1307327

**Published:** 2023-10-24

**Authors:** Rajib Roychowdhury, Carolina Ballén-Taborda, Palak Chaturvedi

**Affiliations:** ^1^ Department of Plant Pathology and Weed Research, Institute of Plant Protection, Agricultural Research Organization (ARO) – Volcani Center, Rishon Lezion, Israel; ^2^ Pee Dee Research and Education Center, Department of Plant and Environmental Sciences, Clemson University, Florence, SC, United States; ^3^ Molecular Systems Biology Lab (MOSYS), Department of Functional and Evolutionary Ecology, Faculty of Life Sciences, University of Vienna, Vienna, Austria

**Keywords:** genetic resources, crop improvement, trait characterization, abiotic stress, disease resistance, multi-omics, CRISPR-Cas, climate resilience

Global food security is a major concern in the ongoing climate-changing scenario; therefore, developing resilient crop cultivars and hybrids is of high priority in this 21^st^ Century to feed an ever-growing population with sufficient and nutritious food. In addition to major crops - rice, wheat, and maize -, it is important to explore orphan/native crops that have more nutritional value (Chaturvedi et al., 2022; Chaturvedi et al., 2023). Biotic stressors including fungi, bacteria, nematodes, insects, and viruses; and abiotic limiting conditions such as drought, heat, cold, salinity, flooding, and nutrient deficiency in soils, have been exacerbated due to climate change (Ghatak et al., 2017; Chaturvedi et al., 2021). Developing and utilizing multiple resilient crops is critical to achieving agricultural seed yields and ensuring food security under all environmental constraints. Raising high-yielding crops under environmental constraints has been daunting due to the low heritability of characters under selection. Identifying more traits of importance that can confer tolerance to various stressors, is the primary goal of scientists and breeders (Roychowdhury et al., 2020). Thus, our Research Topic “*Characterizing and improving traits for resilient crop development*” comprises 14 manuscripts that provide new insights into crop genetic resources, quantitative trait loci (QTL) mapping, genome-wide association studies (GWAS), haplotype analysis, multi-omics approaches, gene discovery, expression and functional characterization using advanced gene editing tools.

Plant diseases cause approximately 30% of yield losses in major crops every year (Gangurde et al.). Under the current climate scenario, many diseases are emerging, worsening crop sustainability in the coming decades (Chakraborty et al., 2014). GWAS have been used to effectively discover QTL associated with resistance to diseases in multiple crop species (Gangurde et al.). Gangurde et al. compiled and highlighted successful GWAS studies reported in the last two decades. Their study mainly focused on improving the power, resolution and efficiency of identifying marker-trait associations through the discovery of important disease resistance genes, major developments in association studies, statistical models and bioinformatic tools. Additionally, it was discussed the challenges and opportunities that GWAS provides for breeding crops. Recently, multi-omics and PANOMICS strategies have been extensively used to uncover genes for marker-assisted breeding (Weckwerth et al., 2020; Roychowdhury et al., 2023). Another review article by Jha et al. described the development of disease-resistant legumes from OMICS integration and plant breeding approaches. Legumes play a crucial role in higher nutrition and as a staple food for farmers in developing and underdeveloped nations. Viral diseases in grain legumes like soybean, mungbean, peanut, chickpea, etc. affect global grain legume production severely. Recently, abiotic stress tolerance in legumes has been evaluated using the OMICS approach (Pazhamala et al., 2020; Jan et al., 2022; Kudapa et al., 2023). The authors have discussed in their review how exploring naturally resistant grain legume genotypes within germplasm, landraces, and crop wild relatives could be a promising, economically viable, and eco-environmentally friendly solution to reduce yield losses (Jha et al.). In peanut or groundnut, foliar diseases (leaf spot and rust) caused by fungal pathogens frequently cause serious yield and quality loss. To incorporate resistance into high-yielding cultivars, Moretzsohn et al. used a wild-derived allotetraploid as a donor in their backcrossing scheme. Moretzsohn et al. used microsatellite markers for foreground and SNP genotyping for background selection and completed GWAS to identify QTL responsible for leaf spot and rust resistance. In such a way, they developed adapted peanut lines with high cultivated genome recovery, improved yield potential, and harbored the resistance QTL. Fusarium height blight (FHB) is a yield and quality-limiting disease of wheat (Boyles et al., 2023). Less FHB incidence has been observed in taller and later-maturity plants (Sari et al., 2020). Cabral et al. conducted a single-locus and multi-locus GWAS using an association mapping panel (AMP). Their study reported 17 quantitative trait nucleotides (QTNs) controlling FHB resistance concerning days to anthesis and plant height, and their corresponding candidate genes. These QTNs and linked SNP markers could be useful in selecting FHB-resistant lines with desired plant height and/or maturity in wheat.

When considering the breeding for tolerance to abiotic stressors, an important trait in wheat is the pre-harvest sprouting (PHS) resistance that causes significant yield losses and is highly influenced by the weather conditions. Considering the changing climate that we are experiencing nowadays; it is imperative to identify QTL controlling PHS. Patwa and Penning conducted a GWAS using a historically diverse set of soft winter wheat genotypes. They have reported 102 genomic regions encompassing 226 QTL controlling PHS, agronomic and flour quality traits. PHS trait-linked QTL has little overlaps with QTL associated with flour quality or agronomic traits, which suggests that it is possible to select PHS resistance without affecting important flour quality or agronomic traits in winter wheat (Patwa and Penning). Another major global cereal - rice production is being limited by various abiotic stressors. In rainfed conditions, rice is generally growing during monsoon and sometimes early flooding creates anoxic conditions in the rhizosphere and hinders coleoptile growth. Trehalose-6-phosphate phosphatase 7 (*OsTPP7*) is responsible for rice anaerobic germination and coleoptile elongation during flooding. In this context, Aung et al. evaluated 475 Korean rice accessions for coleoptile length under normal and flooded conditions to understand their genetic variation, population structure, evolutionary relationships, and functional haplotypes of *OsTPP7*. Most accessions displayed enhanced flooded coleoptile lengths. Haplotype analysis reveals the presence of three cultivated haplotypes showing significant differences in flooded coleoptile length. These findings are a valuable resource for future haplotype-based breeding strategies in rice. In another study, Sheela et al. reported that gene stacking or pyramiding of multiple stress-responsive genes allows rice cultivars to be tolerant to multiple limiting conditions such as moisture stress, salinity, aging, temperature, and oxidative stresses. Through *in-planta* transformation, stress-responsive genes including *Pennisetum glaucum* Heat Shock Factor 4 (*PgHSF4*), *P. glaucum* 47 (*Pg47*), Pea 68 (*p68*), and *Pseudomonas* Aldo Keto Reductase 1 (*PsAKR1*) were co-expressed in rice. The transgenic lines were validated through physio-biochemical traits like chlorophyll content, membrane stability, reactive oxygen species (ROS) accumulation, yield potential, temperature and oxidative stress, and stress-responsive expression of the aforesaid transgenes and stress-responsive genes – *heat shock protein 70* (*HSP70*), *superoxide dismutase* (*SOD*), *ascorbate peroxidase* (*APX*), *late embyogenic abundance 3* (*LEA3*), *salt overlay sensitive 1* (*SOS1*), *protein phosphatase 2C* (*PP2C*) and *proline 5 carboxylase* (*P5CS*). This multigene combination can potentially improve tolerance to multiple abiotic stress conditions and pave the way for developing climate-resilient crops.

Other than the row crops and vegetables, breeding could be extended to other economically and medically important crops like sugar maple, cassava and patchouli. Sugar maple is an economically important tree species for its syrup and hardwood production, and the trees are highly vulnerable to drought conditions. Mulozi et al. characterized physiological and biochemical traits, where chlorophyll biosynthesis, lipid membrane damage, and osmolyte production play a crucial role in responding to drought. Additionally, genome-wide expression profiling using RNAseq allowed the identification of differentially expressed genes (DEGs) involved in stress-responsive pathways. This study provides an understanding of molecular, physiological, and biochemical drought-tolerant mechanisms in sugar maple. Cassava is one of the most important root crops in the world as a staple food crop (De Souza et al., 2017). It is characterized by the accumulation of starch in its root tissue. Pan et al. studied the properties of the 14-3-3 protein to characterize the structures and pathways involved in starch accumulation in cassava roots. High and low-starch-producing cultivars were studied in which *Me14-3-3* genes were highly expressed in high-starch cultivars, while in the late stage of starch accumulation, *Me14-3-3* genes were highly expressed in low-starch cultivars. Based on the transgenic over-expression in *Arabidopsis* and its functional validation in virus-induced gene silencing (VIGS) in the cassava leaves and roots, support the negative effect that *Me14-3-3II* has on starch accumulation. Patchouli is an important medicinal plant for patchouli propagation and oil yield. Warsi et al. studied this drought susceptible plant to establish a rapid and efficient *in vitro* regeneration method followed by agrobacterium-mediated transformation with *ACC deaminase* gene. For tissue culture, they used leaf, petiole, and transverse thin cell layer (tTCL) as explants. Some of the regenerated transgenic plants were drought resistance validated by physiological indicators, oxidative stress, and genes’ relative expression. These findings provided new opportunities for genetic manipulation to achieve drought-resistant patchouli plants for commercial cultivation.

For trait characterization, CRISPR-Cas mediated gene editing has become a useful tool to improve abiotic and biotic stress responses in vegetable crops such as tomatoes and cucumbers (Singh et al., 2020). Tran et al. created gene-edited variants lines of tomato through CRISPR multiplexing. They targeted salt stress negative regulator - *hybrid proline-rich protein 1* (*HyPRP1*) with two functional domains - proline-rich domain (PRD) and eight cysteine-motif (8CM) removed. This loss of function makes tomato more salt (osmotic) tolerance with higher resistance to *Pseudomonas syringae* pv. *tomato*. However, such genetic changes increase the susceptibility to Fusarium wilt. Tek et al. used CRISPR-Cas9 in cucumber inbreed lines susceptible to powdery mildew caused by *Podosphaera xanthii* to knockout the *CsaMLO* function. *CsaMLO* is a negative regulator of powdery mildew disease, and via CRISPR modification on this locus, three mutant lines were generated - *CsaMLO1*, *CsaMLO8*, and *CsaMLO11*. These mutant lines were further validated through the accumulation of ROS as studied by Li et al. (2023) in wheat. The results suggest that the *CsaMLO8* mutation governs pre-invasive resistance, whereas the other two double mutations give hypersensitive response-aided resistance to *P. xanthii* (Tek et al.). In addition, Fidan et al. used CRISPR to edit the *elF4E* gene in cucumber, which acts as a target for potyviruses like watermelon mosaic virus (WMV), papaya ringspot virus (PRSV) and zucchini yellow mosaic virus (ZYMV). They used two inbred lines and targeted 1^st^ and 3^rd^ exons of *elF4E* by using two guide RNAs (gRNA1 and gRNA2) to produce transgene-free plantlets. Further, the plantlets from each inbred line were crossed to produce F1 population to check the allelic effects of *elF4E* mutations. Any disease symptoms are not visualized in homozygous single and double mutants. Therefore these findings describe an effective route for mass production of viral-resistant cucumber to WMV, ZYMV, and PRSV. Translational biology in plant sciences is gaining momentum in crop breeding research in recent years. Son and Park highlighted the translation mechanisms modulated by biotic, hypoxia, heat, and drought stressors and provided a strategy to improve stress tolerance in crops by modulating translational regulation. Cap-dependent and internal ribosomal entry site (IRES)-dependent translation initiation mechanisms are activated during translation reprogramming in response to stress stimulus. Under any pathogenic attack, translational control for pattern-triggered immunity (PTI) and effector-triggered immunity (ETI) provide the major resistance signal in the plant.

The characterization and improvement of traits for resilient crop development must be of high priority in global efforts to address the challenges imposed by climatic change, increasing population, and food security concerns (**Figure 1**). The understanding of traits in crop species, whether related to stress tolerance, yield, disease resistance, or nutritional content, will allow to development of resilient crop varieties and hybrids. This Research Topic highlights the impact of advanced techniques in genomics, molecular biology, and breeding to unravel the genetic basis of traits but also create innovative strategies to enhance crop resilience.

**Figure 1:**
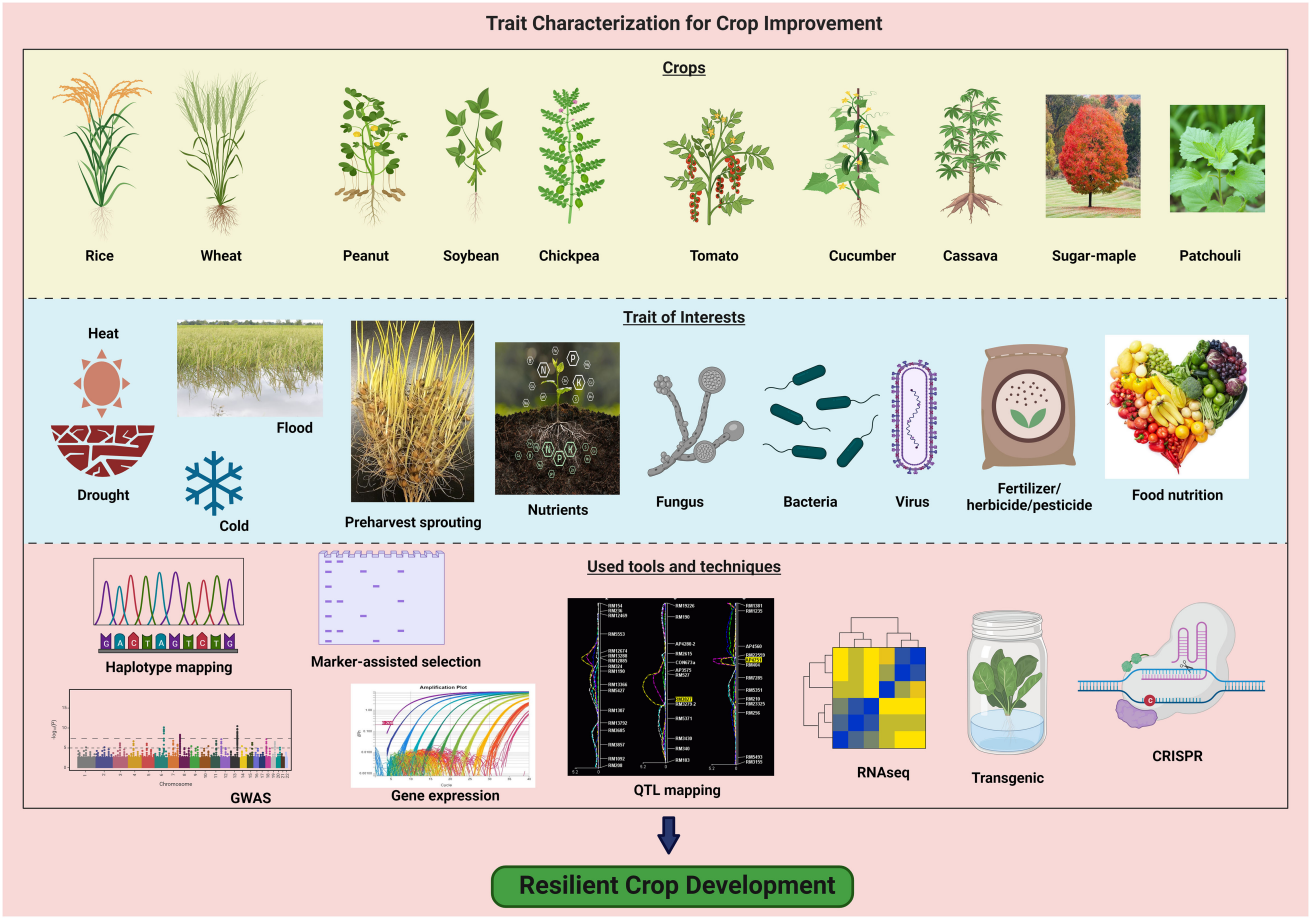
Different crop plants, traits of interest and various genomic tools for trait characterization lead to resilient crop development. The diagram was created using BioRender (https://biorender.com/) premium version.

## Author contributions

RR: Conceptualization, Visualization, Writing – original draft, Writing – review & editing. CB-T: Conceptualization, Writing – original draft, Writing – review & editing. PC: Conceptualization, Writing – original draft, Writing – review & editing.
